# Dynamic Demand Evaluation of COVID-19 Medical Facilities in Wuhan Based on Public Sentiment

**DOI:** 10.3390/ijerph19127045

**Published:** 2022-06-08

**Authors:** Zijing Ye, Ruisi Li, Jing Wu

**Affiliations:** 1 School of Urban Design, Wuhan University, Wuhan 430072, China; 2018302091018@whu.edu.cn (Z.Y.); 2018302091020@whu.edu.cn (R.L.); 2 Hubei Habitat Environment Research Centre of Engineering and Technology, Wuhan 430072, China

**Keywords:** COVID-19, public sentiment, social media, medical facility, dynamic demand evaluation

## Abstract

Medical facilities are an important part of urban public facilities and a vital pillar for the survival of citizens at critical times. During the rapid spread of coronavirus disease (COVID-19), Wuhan was forced into lockdown with a severe shortage of medical resources and high public tension. Adequate allocation of medical facilities is significant to stabilize citizens’ emotions and ensure their living standards. This paper combines text sentiment analysis techniques with geographic information system (GIS) technology and uses a coordination degree model to evaluate the dynamic demand for medical facilities in Wuhan based on social media data and medical facility data. This study divided the epidemic into three phases: latent, outbreak and stable, from which the following findings arise: Public sentiment changed from negative to positive. Over half of the subdistricts in three phases were in a dysfunctional state, with a circular distribution of coordination levels decreasing from the city center to the outer. Thus, when facing major public health emergencies, Wuhan revealed problems of uneven distribution of medical facilities and unreasonable distribution of grades. This study aims to provide a basis and suggestions for the city to respond to major public health emergencies and optimize the allocation of urban medical facilities.

## 1. Introduction

Coronavirus disease (COVID-19) is a new infectious disease that is mainly transmitted by respiratory droplets and contact and is generally infectious to human beings [1]. The impact of the COVID-19 pandemic is the most significant flu pandemic since 1918, which resulted in a huge impact worldwide [2]. According to the World Health Organization, as of 16 February 2022, the total number of confirmed COVID-19 cases globally is 414,525,183, including 5,832,333 deaths [3]. As a result, the COVID-19 epidemic becomes the focus of worldwide attention. Since the outbreak of the COVID-19 epidemic, a large number of relevant studies, mainly in the areas of disease characteristics and causative mechanisms [4,5,6,7,8], public mental health [9,10,11,12], socio-economic impact [13,14,15] and social governance [16,17,18,19], with the medical field dominating, have been conducted by researchers in various fields worldwide. Relatively few studies have been carried out at the spatial level, mainly focusing on the spread pattern of the COVID-19 epidemic between provinces and cities [20,21,22], and the spatial factors that affect the spread of the epidemic. Several studies have mentioned the role of migrant population density [23,24] or lived population density [25,26,27] on the spread of COVID-19, in addition to studies that have explored factors such as Within this scope, medical facilities are an important part of the city’s public facilities, and the effective deployment of medical facilities plays an important role in curbing the rapid spread of COVID-19. Therefore, exploring the allocation of urban healthcare facilities is important in responding to major public health emergencies.

The uncertainty and strong contagiousness of COVID-19 not only threatens one’s physical health but also seriously affects one’s psychological health, especially in terms of one’s emotional well-being [28,29]. An effective way to stop the spread of the epidemic is to stop human communication and activity, which can be extremely emotionally challenging. In China, COVID-19 was first identified and rapidly broke out in Wuhan, Hubei Province [8]. To slow and stop the spread of the virus, Wuhan declared a city lockdown on 23 January 2020, suspending public transport in the city and imposing transport restrictions on individuals [30]. This measure has been described as the most severe epidemic prevention measure ever taken in human history for a major city with a population of 10 million, and the people of Wuhan faced prolonged panic and confusion as a result [31,32,33]. Under this circumstance, a large number of studies on human emotions during the epidemic have emerged, but most of the existing studies have focused on specific groups, such as health care workers, infected patients, or susceptible people [34,35,36], mainly in the areas of mental health [37,38,39] and public reactions [40,41], and only a few studies have focused on the spatio-temporal characteristics of mood fluctuations of home-isolated citizens [42].

At the same time, public mood and the allocation of healthcare facilities have clear correlation [22]. Hospitals, as facilities for daily life services, are conducive to evoking positive public sentiment [43]. However, when faced with a major public health emergency, hospitals are high-risk areas with large numbers of pathogen carriers that can facilitate the spread of the virus amongst health care workers, patients and hospital visitors, thereby leading to negative emotions amongst the surrounding population [2]. Therefore, this study focused on public sentiment and medical facility during the COVID-19 outbreak to explore the concordance between them.

The spread of COVID-19 has led to increased difficulty in on-site social surveys and small, time-consuming research samples [44]. Social media data provide geolocation data with temporal information [45], which creates a new solution for relevant research. With its real-time nature and high interactivity, social media has become an important way for citizens in terms of self-perception and information exchange in the process of controlling the spread of the COVID-19 [46,47,48,49]. These spatiotemporal data have great potential to be used for machine learning and text mining to analyse and understand human behaviour, public reactions, potential courses of action and public opinion better [50,51].

This study analyses public sentiment at different stages of COVID-19 through social media data; dynamically evaluates the allocation of urban medical facilities during the COVID-19 epidemic at a finer spatial scale; and uses a coordination degree model to determine the level of coordination between them, thereby providing a basis and suggestions for future urban responses to severe public health emergencies and optimising the spatial allocation of urban healthcare resources. The respondents of this study are all discussions about COVID-19 on Weibo by Wuhan residents during the rapid spread of COVID-19. The methods of this paper consist of four steps: (1) by using keywords and SVM classifier to filter the microblog text data related to COVID-19 and classify the epidemic into different phases according to public attention; (2) by using an extended sentiment dictionary to determine the sentiment polarity of Weibo text; (3) by analysing the spatial distribution characteristics of public sentiment and medical facilities at different levels during various stages of the epidemic; and by (4) measuring the ratio of sentiment values and medical facility coverage in each subdistrict of Wuhan at different stages of the epidemic and determining the coordination level based on the coordination degree model. The paper is structured as follows: the first part is an introduction and research background. The second part discusses the basic social background of the study area, data sources and pre-processing process. The third section explains the screening method and sentiment polarity determination method of Weibo text, as well as the kernel density method and the coordination model used subsequently. The three latter sections present the results, discussion and conclusions.

## 2. Materials and Methods

### 2.1. Study Area

Wuhan (113°41′–115°05′ E, 29°58′–31°22′ N) has a total area of 8569.15 km^2^ and a resident population of 11,212,000 in 2019 [52]. It is the most populous city in Central China [53] and is the capital city of Hubei Province. Wuhan was the first outbreak area in China where COVID-19 was detected [54]. According to the National Health and Wellness Commission of the People’s Republic of China, as of 8 April 2020, the cumulative number of confirmed COVID-19 cases in Wuhan reached 50,008, accounting for 61% of the total cases in China at that time [55]. Therefore, the selection of Wuhan as the study area is representative and typical.

**Figure 1:**
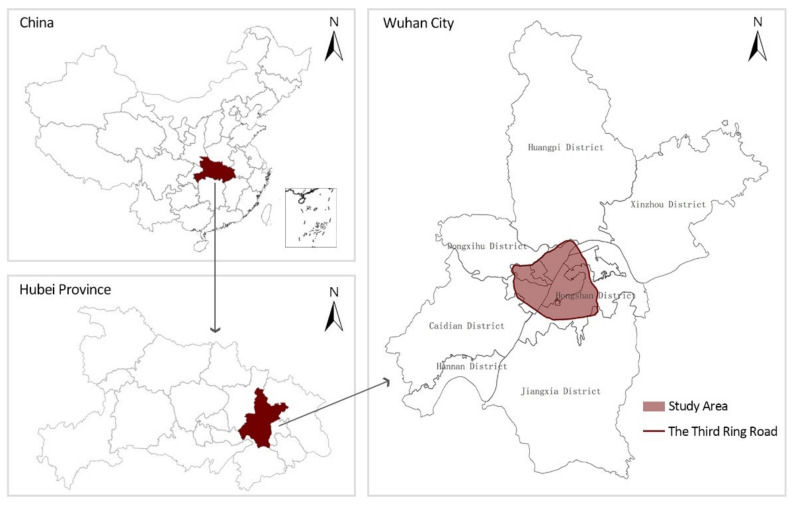
Map of the study area in Wuhan.

Three ring roads are found in Wuhan, and the Third Ring Road is basically the boundary between the city and the suburbs [56]. The Third Ring Road is 91 km long and encircles the entire city centre. The geographical coordinates of medical facilities and Weibo data in Wuhan are all concentrated within the Third Ring Road, so the core area of Wuhan’s Third Ring Road is selected as the study area (Figure 1).

**Figure 2:**
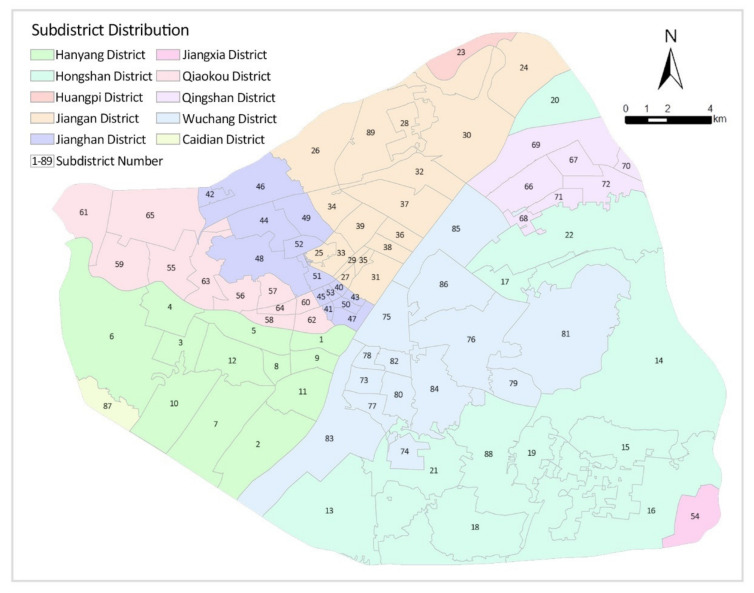
Distribution of subdistricts in the centre of Wuhan.

Wuhan covers an area of approximately 860 square kilometres within the Third Ring Road, covering seven administrative districts, namely, Jiang’an District, Jianghan District, Qiaokou District, Hanyang District, Wuchang District, Qingshan District and Hongshan District, which contains a total of 89 subdistricts (Figure 2). Subdistrict is one of China’s administrative divisions governed by a municipal district, county-level city, county, autonomous county, flag, special district or directly by a prefecture-level city. The governance structure of subdistricts is the subdistrict committee, which is an important part of the political system [57] and played an important role in the fight against the COVID-19 epidemic in China. As such, it is the choice of subdistricts as the basic study unit.

### 2.2. Data Sources

#### 2.2.1. Weibo Data

The social media data used in this study are from the Sina Weibo platform (http://s.weibo.com/weibo/ (accessed on 18 March 2022)). Sina Weibo (Beijing, China, 2009) is one of the most popular social media platforms in China and the most visited Chinese microblog. As of September 2020, the number of monthly active users has reached 511 million, and approximately 224 million people are using Sina Weibo daily. During the COVID-19 period, 6.076 million authoritative information about the epidemic situation were released through Weibo, and the number of readings by residents reached 364.7 billion. A total of 30,000 live broadcasts of the epidemic were launched, with more than 3 billion viewers [58]. Therefore, Weibo is an appropriate setting to observe the emotional changes brought by the COVID-19 outbreak [59]. The data used in this study include the text content posted by the user, the geographical location and the time of posting. Considering that the date of diagnosis of the first COVID-19 patient in Wuhan dates back to 1 December 2019 [8] and that the city was lifted from closure on 8 April 2020 [60], the Weibo texts from that time period were selected for the study. Data that were incomplete and outside the study area were cleaned up, thereby resulting in a total of 68,478 original Weibo text data from the study area. All texts data in relation to COVID-19 were obtained after being filtered by the SVM classifier, which will be described in Section 2.3.1 in detail.

#### 2.2.2. Medical Facilities Data

In China, the majority of healthcare facilities consist of public medical facilities, which are therefore the focus in this study. This study classifies public medical facilities during the COVID-19 epidemic into seven types: tertiary hospitals, secondary hospitals, primary hospitals, community clinics, drugstores, Fangcang shelter hospitals and COVID-19 designated hospitals. According to the “Basic Standards for Medical Institutions (for Trial Implementation)” [61] promulgated by the former Chinese Ministry of Health (now known as the National Health Commission of the People’s Republic of China), primary hospitals include community and township health centres, which provide preventive, medical and rehabilitation services directly to the public. Secondary hospitals provide comprehensive medical services to the region and undertake some teaching and research tasks. Tertiary hospitals provide a high level of specialist medical services and undertake advanced teaching and research tasks [62]. Community clinics provide public health and primary care services primarily to members of the community. Drugstores are facilities that sell medicines and provide basic dispensing services. Fangcang shelter hospitals are large temporary hospitals built by converting public venues, such as stadiums and exhibition centres, to isolate patients with mild-to-moderate symptoms of infectious diseases from their families and communities whilst providing medical care, disease surveillance, food, shelter and social activities [63]. The COVID-19-designated hospitals are those established for the treatment of suspected and confirmed cases of COVID-19 infection.

**Figure 3:**
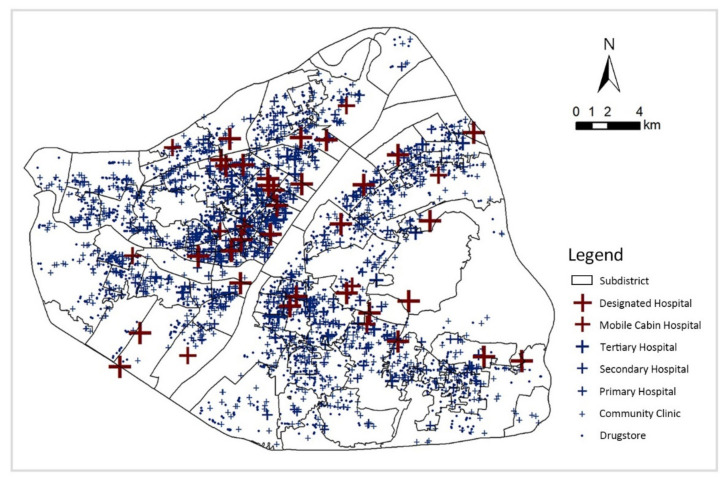
Distribution of seven types of medical facilities in Wuhan.

This study obtained data on medical facilities in Wuhan, including name, level, category and geographical location through Wuhan Municipal Health Commission (http://wjw.wuhan.gov.cn/ (accessed on 18 March 2022)). A total of 40 tertiary hospitals, 52 secondary hospitals, 187 primary hospitals, 1382 community clinics, 2247 pharmacies, 9 Fangcang shelter hospitals and 32 COVID-19 designated hospitals were obtained within the third ring of Wuhan (Figure 3).

### 2.3. Methods

#### 2.3.1. Extraction of Weibo Data Related to COVID-19

Support vector machines (SVM), which are effective classification and regression methods [64], can identify the relevance of text to events based on their content. Therefore, this paper uses an SVM classifier to filter the data. In the classification, Weibo texts unrelated to COVID-19 are several times more frequent than related ones. Thus, filtering the Weibo text using the high-frequency words related to COVID-19 obtained through word frequency statistics is necessary before classification [65]. In this study, 69 high-frequency words related to the COVID-19 epidemic, including ‘Lockdown’, ‘Pneumonia’ and ‘Diagnosed’, were screened for keywords mentioned more than 250 times in the text of Weibo between 1 December 2019 and 8 April 2020 (Table 1), and 30,689 related Weibo texts were obtained after screening. SVM was then used for classification, and 1204 texts were randomly selected as training data for manual labelling, of which a total of 770 were labelled as relevant to the event, resulting in the extraction of 23,891 texts related to the COVID-19 epidemic.

#### 2.3.2. Determination of the Sentimental Polarity of Weibo Texts

Once the COVID-19-related Weibo texts have been filtered, the next step is to determine the emotional polarity of the text. As far as Chinese emotion dictionary resources are concerned, How Net is a relatively comprehensive knowledge base, and this study builds on it by supplementing it with typical Weibo emotion words to establish a Weibo emotion dictionary. The Weibo text included words and emoticons, and for accurate calculation, the emoticons were transformed into corresponding text and incorporated into the emotion lexicon (e.g., [like] [heart] were added to the positive emotion words, and [tear] [sad] were added to the negative emotion words (Table 2)).

A short script in Python 3.8.3 was used to clean up the Weibo text, it cleans out all symbols and English characters, leaving only Chinese characters, and jieba segmentation tool was applied to split the text into emotion words, adverb of degree words and negative words. Positive emotion words have a value of 1, and negative emotion words have a value of −1. Different adverb of degree words in the text indicates various levels of emotional strength [66], and the weights of each adverb of degree word are presented as follows (Table 3).

Retrieve whether there is an adjacent negative word with the sentiment word, and if not, calculate its sentiment score according to the adverb of degree word, the algorithm is presented as:

(1) $$M_{wi}=R_{wa}\times S_{wi}$$

$M_{wi}$ denotes the score of sentiment words, $R_{wa}$ denotes the weight of adverb of degree words, and $S_{wi}$ denotes the weight of sentiment words.

If an adjacent negation exists, then the position of the negative word and the adverb of degree word needs to be determined firstly. When a negative word precedes an adverb of degree word, it indicates a weakening of the emotion, and $M_{wi}$ is assigned to 0.5$S_{wi}$. When a negative word follows an adverb of degree word, it indicates the opposite emotion, and $M_{wi}$ is assigned to −2$S_{wi}$. 

Finally, the total score obtained by accumulating all steps is the emotional polarity of the text, and the algorithm is:

(2) $$Value\left(D_{j}\right)=\sum M_{wi}$$

(3) $$Value\left(D_{j}\right)\left\{\begin{array}{c} >0;\;positive\;emotion\\ =0;\;neutral\;emotion\\ <0;\;negative\;emotion \end{array}\right.,$$

where $Value\left(D_{j}\right)$ denotes the emotional polarity of the Weibo text.

#### 2.3.3. Kernel Density Analysis of Sentimental Points and Medical Facilities

Each text is presented as a point on a map according to its geographical coordinates, and each point has a corresponding emotional polarity, which is referred to as an emotional point in this paper [67]. To further analyse the spatial distribution characteristics of emotional points and medical facilities, the kernel density method was used to process the two sets of spatial data. Kernel density analysis can calculate the unit density of the measured values of points and line elements within a specified neighbourhood, intuitively reflecting the distribution of discrete measured values in the continuous area, which can be used for disease transmission risk prediction and spatial distribution pattern analysis [68,69]. The function is expressed as follows:

(4) $$\hat{f_{h}}\left(x\right)=\frac{1}{nh}\sum_{i=1}^{n}K\left(\frac{x-x_{i}}{h}\right),$$

where *K* denotes the kernel (non-negative function), *h* > 0 denotes the smoothing parameter of the bandwidth, *xi* denotes the sampling point.

#### 2.3.4. Measurement of the Coordinated Relationship between Public Sentiment and Medical Facilities

Coordinated development theory is often used to study the allocation of public service facilities in cities. This study will determine the coordination relationship between public sentiment and medical facilities at the subdistrict scale based on the coordination model.

Firstly, the sentimental polarity of all emotional points within each subdistrict was averaged separately to obtain their combined sentimental values, with the following functional expressions:

(5) $$Value\left(S_{o}\right)=\frac{1}{n}\sum Value\left(D_{j}\right),$$

where $Value\left(S_{o}\right)$ denotes the sentiment value, *n* denotes the number of Weibo texts, and $Value\left(D_{j}\right)$ represents the sentiment value of each Weibo texts.

Secondly, the radiation radius of medical facilities at all levels is set in accordance to the “Standard for urban residential area planning and design” [70], and corresponding buffer zones are constructed for medical facilities of different levels (Table 4).

The area covered by different levels of medical facility buffers in each subdistrict are determined, the covered areas are added up, and the ratio of the total covered area to the area of the subdistrict, which is the coverage ratio of medical facilities in each subdistrict, is calculated with the following functional expressions:

(6) $$M=\sum S/S_{c},$$

where *M* denotes the coverage ratio of medical facilities, *S* indicates the sum of the area where the subdistrict intersects the buffer zone of each medical facility, and $S_{c}$ denotes the area of each subdistrict.

Finally, the coordinated development degree of the two is calculated, and the expression of the coordination degree model function is as follows:

(7) $$C={\left\{\frac{f\left(x\right)\times g\left(y\right)}{{\left[\frac{f\left(x\right)+g\left(y\right)}{2}\right]}^{2}}\right\}}^{k},$$

(8) $$T=\alpha f\left(x\right)+\beta g\left(y\right),$$

(9) $$D=\sqrt{C\times T},$$

where *C* is the coordination degree, taking the value range from 0 to 1; *f*(*x*) and *g*(*y*) are the sentiment value and medical facility coverage ratio of each street, respectively; *k* is the adjustment coefficient, taking the value of 2 in this study; *T* is the comprehensive rating index; *α* and *β* are the weights; the study considers emotion and medical facilities equally important, so *α* and *β* are taking the value of 0.5; and *D* is the coordination development degree, its value is from 0 to 1, and the larger the value the better the coordination. In this paper, the degree of coordinated development is classified into 10 levels with reference to relevant studies [71,72] (Table 5).

## 3. Results

### 3.1. Trend in Public Attention of COVID-19 Epidemic

**Figure 4:**
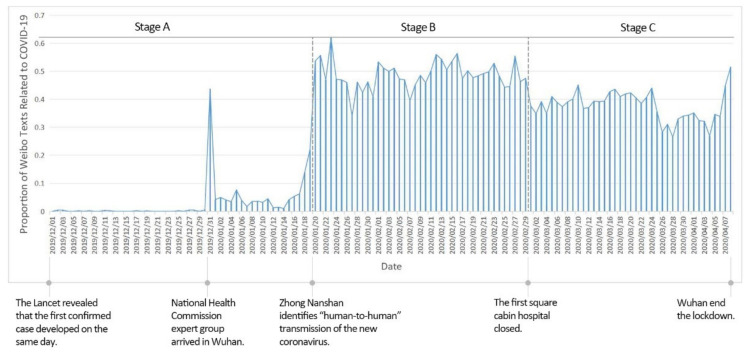
Trend in public attention of COVID-19-related Weibo texts.

The COVID-19 epidemic is divided into three phases based on the daily public attention to the epidemic (i.e., the number of relevant Weibo texts as a percentage of the total for the day (Figure 4)): Stage A (1 December 2019 to 19 January 2020) was the incubation period, with low and unstable public attention. There was a spike in public attention on 31 December 2019 due to the arrival of a team of experts from the China Health Care Commission in Wuhan to investigate an unidentified pneumonia, before the heat dropped back. Stage B (20 January to 29 February 2020) is the explosive period, during which public attention rising rapidly, and the heat staying high, with the daily percentage of relevant Weibo texts at around 50%. There were many peaks and fluctuations in public concern during this phase, with the highest value occurring on 23 January 2020, the day of the Wuhan lockdown. Stage C (1 March to 8 April 2020) was the stabilization period, when public attention to the COVID-19 epidemic stabilized, with the daily percentage of relevant Weibo texts at around 40%. Overall, public attention to the epidemic in Wuhan ranged from low to high, with a rapid outbreak on 20 January 2020 that stabilized at a later stage.

### 3.2. Trend in Sentimental Polarity

**Figure 5:**
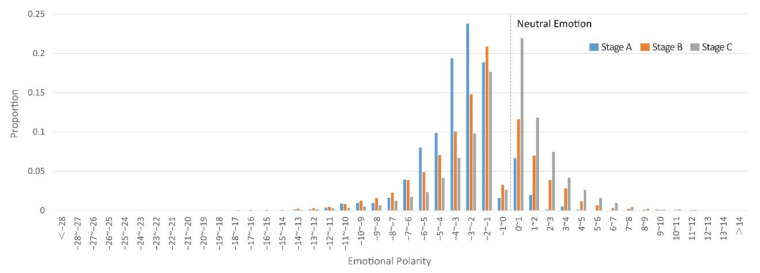
Distribution of sentimental polarity for the three stages.

Considering 3191 Weibo texts with neutral sentiment, accounting for only 13% of the total, and most of them were news reports, which could not reflect public sentiment, this part of the Weibo texts was excluded. After determining the polarity of the sentiment, the distribution of the scores of the emotional points at each stage is detailed as follows (Figure 5). In Stage A, negative emotions accounted for 90%, and the largest number of emotional points in the range of −3 to −2 was 24%. In Stage B, negative emotions accounted for 72%, and the largest number of emotional points in the range of −2 to −1 was 21%. In Stage C, negative emotions accounted for 48%, and the largest number of emotional points in the range of 0 to 1 was 22%. The proportion of negative emotions gradually decreases in the three stages, and there is an overall trend of change from negative to positive.

**Figure 6:**
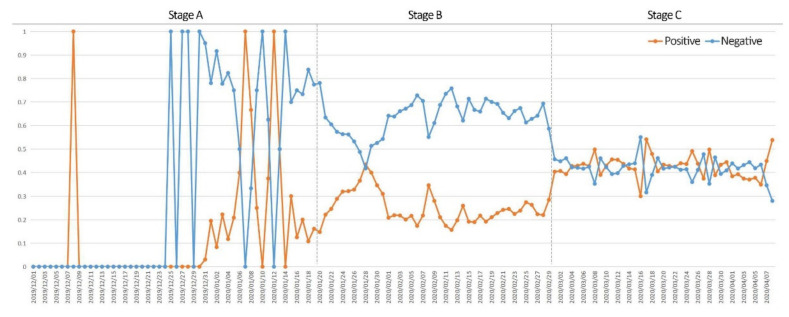
Positive and negative sentiment trend.

To understand the specific changes in sentiment during the COVID-19 outbreak and to investigate the reasons for these changes, the daily percentages of positive and negative public sentiment were calculated for the three phases of the COVID-19 outbreak (Figure 6). In Stage A, positive and negative emotions fluctuate and are unstable. In Stage B, as the COVID-19 outbreak spread in Wuhan, the proportion of negative sentiment was significantly greater than that of positive sentiment. In Stage C, the proportion of positive and negative sentiments on related Weibo texts tended to be balanced, and the sentiment fluctuated less. The reason is that with the passage of time and the improvement in information, public awareness of the COVID-19 epidemic gradually increased, and the establishment of related medical facilities in Wuhan, such as COVID-19-designated hospitals and Fangcang shelter hospital, enabled the epidemic to be effectively controlled, thereby reducing the magnitude of public sentiment fluctuations.

### 3.3. Spatial Distribution of Emotional Points and Medical Facilities

#### 3.3.1. Kernel Density of Emotional Points

**Figure 7:**
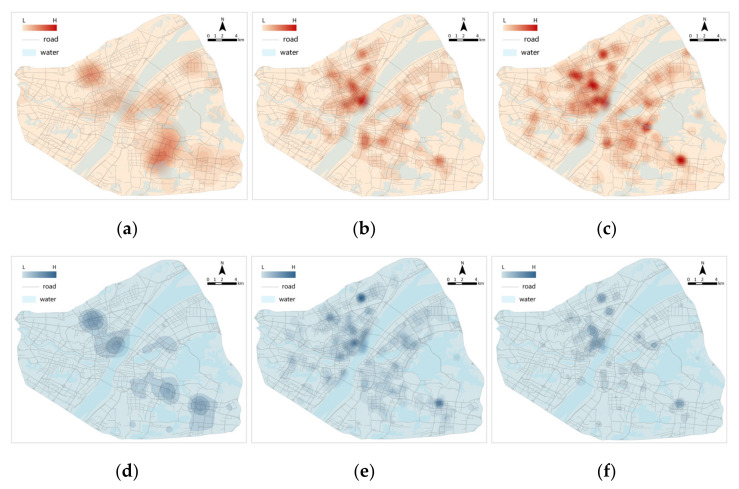
Kernel density distribution of emotional points: (**a**) positive emotional points in Stage A; (**b**) positive emotional points in Stage B; (**c**) positive emotional points in Stage C; (**d**) negative emotional points in Stage A; (**e**) negative emotional points in Stage B; (**f**) negative emotional points in Stage C.

The information on the geographical coordinates of the emotional points was used to further explore their spatial distribution. Using the kernel density analysis method, the kernel density distribution of positive and negative emotional points in the three stages was obtained (Figure 7). In Stage A, positive and negative emotions were mostly concentrated in the districts of Jianghan, Jiang’an and Wuchang. In Stage B, the number of positive and negative emotions increased. Negative emotions increased extensively but were still concentrated in the districts of Jianghan, Jiang’an and Wuchang. In Stage C, positive sentiment continued to increase and was widespread. Negative sentiment tends to contract and diminish in intensity. This finding is highly consistent with the spread of the COVID-19 epidemic in Wuhan.

#### 3.3.2. Kernel Density of Medical Facilities

In response to the COVID-19 epidemic, Wuhan took measures, such as setting up COVID-19-designated hospitals and building Fangcang shelter hospitals, so the distribution of medical facilities at different stages in Wuhan varied during the COVID-19 epidemic (Table 6).

**Figure 8:**
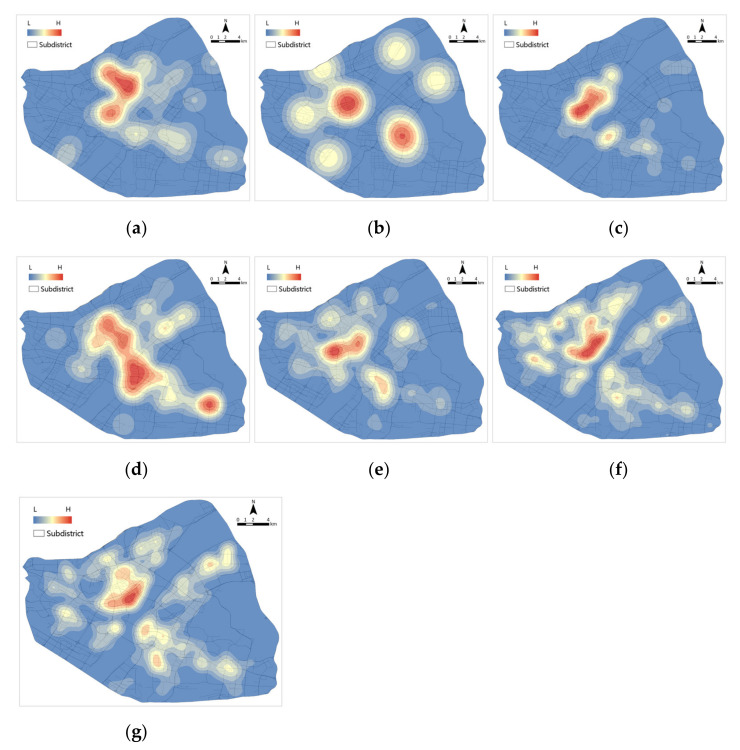
Kernel density distribution of medical facilities: (**a**) COVID-19-designated hospital; (**b**) Fangcang shelter hospital; (**c**) tertiary hospital; (**d**) secondary hospital; (**e**) primary hospital; (**f**) community clinic; (**g**) drugstore.

A kernel density analysis of the distribution of medical facilities at all stages in Wuhan between 1 December 2019 and 8 April 2020 (Figure 8) shows that the distribution of medical facilities in Wuhan is relatively even, with the richest areas for all types of medical facilities concentrated in the central city. The distribution of tertiary hospitals and COVID-19-designated hospitals is uneven, mainly concentrated in the districts of Jianghan and Jiang’an. Community clinics and drugstores are the most widely distributed, followed by primary hospitals, secondary hospitals and Fangcang shelter hospitals.

### 3.4. Coordinated Development Level Evaluation of Public Sentiment and Medical Facilities

#### 3.4.1. Sentimental Value Distribution

**Figure 9:**
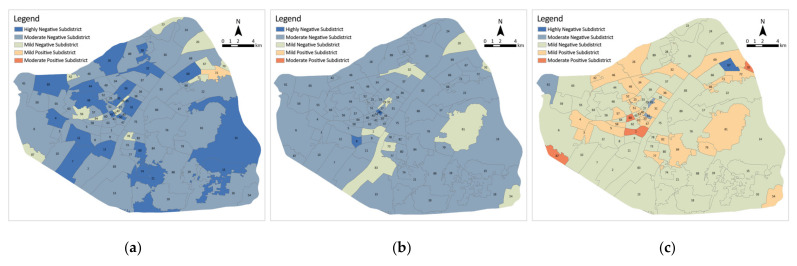
Distribution of sentimental values on a subdistrict scale: (**a**) Stage A; (**b**) Stage B; (**c**) Stage C.

To reflect the spatial distribution of public sentiment in detail, it was implemented into the subdistrict scale for exploration. The composite sentiment value of each subdistrict was obtained by averaging all sentiment points, and the subdistricts were classified into six categories according to the composite sentiment value (Figure 9). Subdistricts with a score greater than 3 were classified as highly positive, 1 to 3 as moderately positive and 0 to 1 as mildly positive, less than −3 were classified as highly negative, −3 to −1 as moderately negative and −1 to 0 as mildly negative. Moderately negative subdistricts accounted for the largest number of subdistricts with 53 (59.6%), followed by highly negative subdistricts with 19 (21.3%), mildly negative subdistricts and mildly positive subdistricts with 4 (4.5%) and 13 (14.6%), respectively, and no moderately positive subdistricts or highly positive subdistricts. In Stage B, the number of highly negative subdistricts decreased to 2 (2.2%), whilst moderately negative subdistricts increased by 23 to 76 (85.4%), mildly negative subdistricts increased to 8 (9.0%). As for positive subdistricts, the number of mildly positive subdistricts decreased to 3 (3.4%), and no moderately positive subdistricts or highly positive subdistricts were recorded, with an overall negative trend. The overall emotion was more positive in Stage C, and the number of positive subdistricts reached 38 (42.7%), but the number of mildly negative subdistricts still accounted for the most, with 50 (56.2%). The number of moderately negative subdistricts dropped to 4 (4.5%), and the number of highly negative subdistricts decreased to 0. The positive sentiment gradually increases, and the negative sentiment gradually decreases in the three stages. No highly positive sentiment was observed in the overall sentiment, and the overall sentiment is all on the negative side, but the positive subdistricts gradually increase as the stages change.

#### 3.4.2. Medical Facility Coverage Ratio Distribution

**Figure 10:**
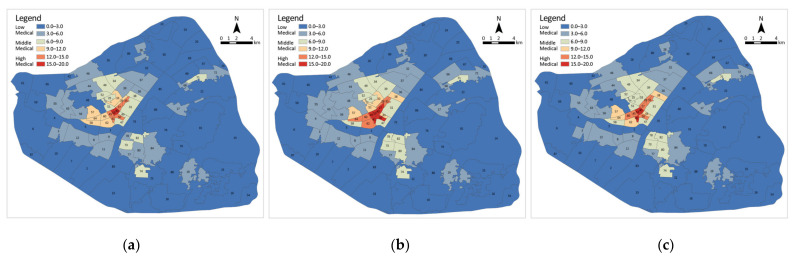
Distribution of the coverage ratio of medical facilities on a subdistrict scale: (**a**) Stage A; (**b**) Stage B; (**c**) Stage C.

Given that the presence of medical facilities varied during the different phases of the epidemic, the ratio of medical facility coverage was measured separately for each subdistrict in the three phases and classified as high, medium or low (Figure 10). Of these, a score greater than 12 is classified as a high medical subdistrict, 6 to 12 as a medium medical subdistrict and 0 to 6 as a low medical subdistrict. In Stage A, there are 62 low medical subdistricts (69.7%), 20 medium medical subdistricts (22.5%) and only 7 high medical subdistricts (7.9%). In Stage B, the number of low medical subdistricts decreased by 3 to 59 (66.3%); medium medical subdistricts decreased to 19 (21.3%); and high medical subdistricts increased to 11 (12.4%). In Stage C, the number of low medical subdistricts remains at 59; medium medical subdistricts have increased to 21 (23.6%); and high medical subdistricts have decreased to 9 (10.1%). The subdistrict with the largest coverage ratio of medical facilities in Stage A is Shuita Subdistrict in Jiang’an District, and in Stage B and C is Qianjin Subdistrict in Jianghan District. In all phases, the high medical subdistricts are concentrated in the inner circle of the city centre, the medium medical subdistricts surround them in the middle circle and the low medical subdistricts are scattered in the outer circle.

#### 3.4.3. Coordinated Development Level Evaluation

**Figure 11:**
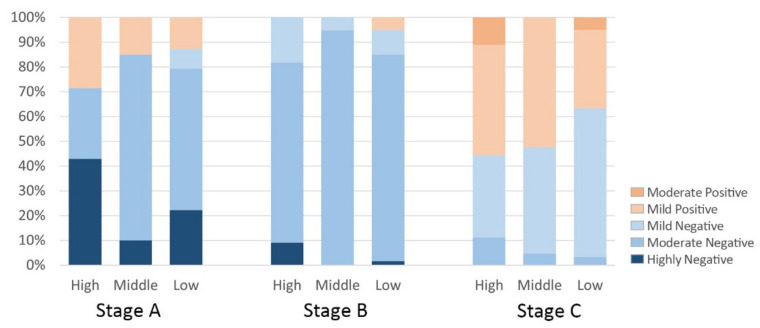
Percentage of each type of sentiment in high, medium and low medical subdistricts.

The sentiment values for each subdistrict were combined with the medical facilities coverage ratio to explore the association and its phase change. Firstly, the percentage of each type of emotion in high, medium and low medical subdistricts was counted in stages (Figure 11). In Stage A, high negative emotions account for the largest share of high medical subdistricts at 42.9% and the smallest share of medium medical subdistricts at 10%, whilst mild positive emotions account for the largest share of high medical subdistricts at 28.5% and the smallest share of low medical subdistricts at 12.9%. In Stage B, there were no positive emotions in the high medical subdistrict and medium medical subdistrict, and highly negative emotions accounted for the largest proportion of the high medical subdistrict at 9.1%. In Stage C, negative emotions accounted for the largest share of low medical subdistricts at 62.7% and positive emotions accounted for the largest share of high medical subdistricts at 55.6%. The results show that the high level of negativity in the subdistricts gradually decreased as the COVID-19 epidemic progressed. In Phases A and C (i.e., the latent and stable phases of the epidemic, respectively), residents’ sentiment is more positive in subdistricts with a larger coverage ratio of health facilities because subdistricts with dense health facilities are better in meeting residents’ access needs and boosting positive public sentiment. In contrast, in Stage B, the outbreak period, residents of subdistricts with large coverage ratios of medical facilities were instead more negative, presumably because hospitals had large concentrations of pathogen carriers, thereby putting nearby residents at higher risk of infection and negatively impacting public sentiment.

**Figure 12:**
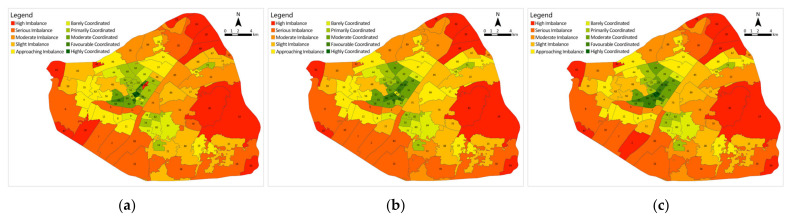
Coordinated development level assessment of subdistricts: (**a**) Stage A; (**b**) Stage B; (**c**) Stage C.

Secondly, the coordination degree model was applied to normalise the sentiment values of each subdistrict, as well as the medical facility coverage ratio and to calculate the coordination development degree to obtain the coordination level of each subdistrict (Figure 12). Stage A contains two highly coordinated subdistricts and three favourable coordinated subdistricts; a total of 33 subdistricts are coordinated (37.1%), mostly located in the city centre in the districts of Jianghan, Jiang’an and Qiaokou. There are also 56 subdistricts that are dysfunctional (62.9%), containing 12 high-imbalance subdistricts, mostly located near the Third Ring Road and in more remote locations. Stage B has a total of 36 subdistricts coordinated (40.4%), with the number of favourable coordinated subdistricts increasing to 8 and no highly coordinated subdistricts. A total of 53 subdistricts are dysfunctional (59.6%), with the number of high-imbalance subdistricts decreasing to 9. Chezhan Subdistrict going from high imbalance to favourable coordinated. In Stage C, the number of coordinated subdistricts recovered to 33 (37.1%), containing 2 highly coordinated subdistricts and 8 favourable coordinated subdistricts. The number of dysfunctional subdistricts increased to 56, containing 12 high-imbalance subdistricts. The distribution of all types of medical facilities in Wuhan, especially tertiary hospitals, varies greatly amongst districts, with most being located in the city centre in the districts of Jianghan, Jiang’an and Qiaokou.

**Figure 13:**
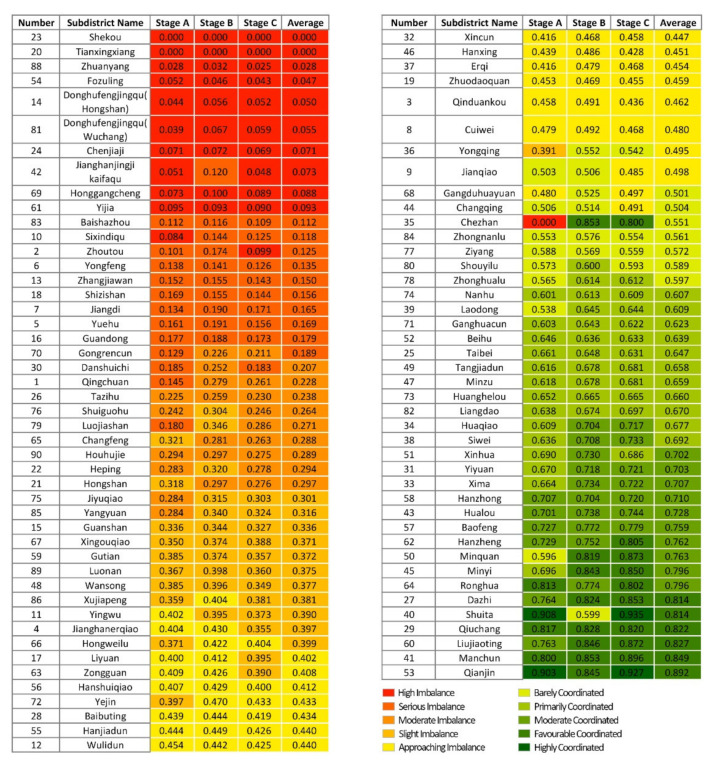
Summary of the coordinated development degree of subdistricts.

The degree of coordinated development of each subdistrict in the three phases is summarised (Figure 13). In comparison with Stage A, 28 subdistricts in Stage B have increased in coordination level, 55 subdistricts have remained the same, and 6 subdistricts have decreased in coordination level. Over 30% of the subdistricts were upgraded in terms of coordination level because during Stage B, the outbreak period, additional medical facilities, such as Fangcang shelter hospitals and COVID-19-designated hospitals, were opened in Wuhan, and the coverage ratio of medical facilities in each subdistrict increased. However, 10% of the subdistricts showed a decline in coordinated development instead, namely, Changfeng Subdistrict, Hongshan Subdistrict, Yingwu Subdistrict, Ronghua Subdistrict, Shuita Subdistrict and Qianjin Subdistrict, where residents’ sentiment was more negative than in Stage A, indicating that the allocation of medical facilities in these subdistricts lagged behind the medical needs based on public sentiment. Compared with Stage B, four subdistricts in Stage C have increased in coordination level, 70 subdistricts have remained the same, and 15 subdistricts have decreased in coordination level. The subdistricts that have decreased in coordination level are mainly located in Wuchang District and near the Third Ring Road. The reason is that as the epidemic was effectively controlled, the Fangcang shelter hospitals were closed one by one, the number of medical facilities was reduced, and the coverage ratio of medical facilities in the subdistricts was reduced. Although public sentiment tends to be positive and stable, the coordination rating has instead declined, indicating that medical facilities in these areas are under-provisioned.

In general, the coordination level shows a circular distribution pattern from the city centre to the periphery, with subdistricts of high coordination level distributed in clusters in Jianghan District, Jiang’an District and Qiaokou District in the city centre (i.e., areas with a high level of medical facilities and a dense distribution), whilst subdistricts with a low coordination level are located near the third ring road and in less inhabited scenic areas (i.e., areas with weak medical resources). More than 50% of the subdistricts in all three stages are in a state of dysfunction, and Stage B is more coordinated than Stages A and C. This finding indicates that there is a deficiency in the allocation of medical facilities in Wuhan, and that increasing medical facilities can alleviate negative public sentiment effectively.

## 4. Discussion

The COVID-19 outbreak prompted researchers to begin rethinking urban space. From historical experience, urban planning is an important way for local governments to safeguard urban public health and reduce infectious diseases. For example, the Black Death epidemic led to the strengthening of basic sanitation facilities in European countries. The cholera epidemic forced the UK government to set a number of health standards and provide public health services [22]. Therefore, creating healthier urban spaces in the post-epidemic era is particularly important.

The key to a healthy city is urban healthcare facilities, which are mostly evaluated in existing studies focusing on spatial accessibility, equity [73,74,75] and supply-demand balance of healthcare facilities [76,77,78]. The outbreak of COVID-19 has reduced the geographic accessibility of medical facilities [79,80,81,82], and the reduction is greater in the urban periphery than in the central city [83]. Different from them, this study focuses on public emotions and evaluates the spatial configuration of medical facilities. it was found that the allocation of medical facilities in Wuhan showed a gradually decreasing distribution pattern from the centre to the periphery, which is similar to other Chinese cities, such as Zhengzhou, Shanghai, and Shenzhen [73,84,85], implying that the government needs to take seriously the lag in healthcare service facilities during urban expansion. On the other hand, public sentiment surveys are highly valuable for improving health systems [86], which is mainly used to evaluate the quality of services provided by the health care system [87,88]. Studies at the spatial level have focused on the urban perspective [22,89], while there is a lack of evaluation of the configuration of health care facilities at a finer scale, this study examines the subdistrict scale to fill this gap.

Nevertheless, this study has limitations, and future refinements are needed in the following areas.

(1) Weibo users are not representative of all Wuhan residents during the COVID-19 pandemic. According to the 2020 Weibo User Development Report [58], most Weibo users are between the ages of 20 and 30, accounting for 48% of the population. In contrast, according to the Wuhan Statistical Yearbook 2021 [90], the proportion of 20- to 30-year-olds in the total population of Wuhan is 14%. The way the information is shared may vary by gender and age. Since few of the social media users are elderly or children, social media data cannot fully reflect public sentiment. Further research considering the diversity of data sources is needed to obtain more extensive and accurate conclusions. In addition, given that there is evidence to suggest that cultural background and household situation can influence perceptions and experiences with the health system [91,92], we will aim to include such questions in similar future surveys.
(2) Due to the diverse forms of expression in Chinese, the use of emotion dictionaries to determine the emotional polarity of text cannot fully identify the emotion expressed by the user, and future research could use methods, such as machine learning, to capture emotion accurately.
(3) Many factors can affect the evaluation of the configuration of medical facilities. Besides considering public sentiment, there are some other variables that cannot be ignored, such as urban population distribution, spatial accessibility, urban spatial development level, actual medical behaviour of residents, spatial spread of COVID-19 epidemics, etc. In the future research, we will further improve the methods and ideas, explore the influence of other factors and the relationship between them, and provide decision reference for the fine layout and function enhancement of perfect urban medical facilities.

## 5. Conclusions

This study analysed the evolution of public sentiment during the rapid spread of COVID-19 in Wuhan using social media data processed by an SVM classifier; explored the relationship between public sentiment and medical facilities at the subdistrict scale; and conducted a detailed dynamic demand evaluation of COVID-19 medical facilities in Wuhan based on public sentiment.

The results show the following: Firstly, public sentiment has an overall positive trend as the phase of the COVID-19 epidemic changes. Public attention to the epidemic ranged from low to high, with a rapid outbreak after the lockdown of Wuhan and a stabilisation at a later stage. This indicates that public sentiment becomes increasingly positive as the epidemic is effectively controlled and as urban medical resources are replenished. Secondly, during the latent and stable stages of the COVID-19 epidemic, residents of subdistricts with a large coverage ratio of medical facilities had more positive sentiments, whilst an opposite pattern is observed during the outbreak. Adequate medical facility allocation was found to elicit positive public sentiment, but dense medical facilities acting as a source of viral infection at specific times can elicit negative public sentiment, so a reasonable grade allocation with moderate density is key to medical facility layout. Thirdly, in all three phases, more than 50% of the subdistricts are in a state of dissonance, and Stage B is more coordinated than Stages A and C. The distribution of coordination levels is consistent across the three phases, thereby showing a circular decreasing pattern from the city centre to the periphery, with subdistricts of high coordination level distributed in clusters in the city centre with a high level of medical facilities and a dense distribution; subdistricts with a low coordination level are located near the third ring road and in less inhabited scenic areas with weak medical resources. It shows that in the face of severe public health emergencies, Wuhan has the problems of uneven distribution of numbers, unreasonable distribution of levels, and insufficient coverage of tertiary hospitals, and the configuration of medical facilities in the periphery of the city is weaker than that in the central area, which provides a basis for future research on the optimization of the spatial layout of medical facilities in Wuhan.

In the future, urban planners should pay more attention to areas with less coverage of tertiary hospitals, such as Wuchang District, and increase the number of medical facilities, especially hospitals, in these areas. For areas with relatively dense medical facilities, a reasonable layout and rational allocation of hospitals is needed to avoid redundancy of resources. Government departments should establish emergency response plans for major public health emergencies, such as setting up temporary hospitals in areas where medical resources are scarce and setting up designated hospitals in their vicinity to safeguard the public’s demand for medical treatment and alleviate negative public sentiment. In addition, community medical facilities play a vital role in the control of the COVID-19 epidemic [93], and should respond to the advocacy of “Building a 15 min Community Life Circle” [94], strengthen the construction of primary healthcare system, improve the level of medical services in community hospitals, and convenience residents by enabling access to medical services closer to home.

The representative characteristics of Wuhan, a metropolis with developed transportation, dense population, and the first city in China to detect COVID-19 virus and its rapid spread, make the research results in Wuhan universal and forward-looking, and may provide lessons for other cities facing major public health emergencies. It is of great value to those involved in urban planning and design and may help them to quickly determine the evolution of public sentiment under a health emergency and whether medical facilities in various areas of the city meet public demand, so that they can act in advance to fill the shortcomings of the urban health system and optimize the spatial layout of urban medical facilities. Additionally, we focus on urban space at the subdistrict scale, which supports the development direction of more humane and refined urban planning in the future.

## Figures and Tables

**Table 1:** Summary of high-frequency words related to the COVID-19 epidemic.

Word	Length	Word	Length	Word	Length
lockdown	1	seek help	2	fever	1
pneumonia	1	severe illness	2	hospitalize	1
diagnosed	1	high temperature	2	cough	1
mask	1	disinfect	1	stranded	1
quarantine	1	nucleic acid	2	governance	1
case	1	suspected	1	infected person	2
patient	1	cold	1	contagion	1
infect	1	heal	1	health commission	2
lift lockdown	2	hospital bed	2	investigate	1
virus	1	receive	1	rehabilitate	1
new	1	health care	2	epidemic area	2
supplies	1	donate	1	asymptomatic	1
fangcang	1	stay at home	2	Huanan	1
medical staff	2	Vulcan hill	2	designated hospital	2
reopen	1	symptom	1	angel in white	3
coronavirus	1	suspected cases	2	Zhong Nanshan	2
prevention	1	treat	1	close contact	2
medical team	2	protective clothing	2	alcohol	1
support	1	epidemic prevention	2	aid	1
anti-epidemic	1	medical personnel	2	battle	1
medical	1	illness	1	Sars	1
test	1	combating epidemic	2	lift a ban	3
protect	1	rush to the rescue	4	bailout	1

**Table 2:** Examples of emotion words.

Item	Examples
Positive emotion words	Come on, salute, happy, moving, [like], [heart]
Negative emotion words	Angry, reluctant, ignore, heartache, [tear], [sad]

**Table 3:** Examples of negative word and adverb of degree word weights.

Item	Weight	Examples
Negative words	−1	No, abandon, oppose, forbid
Adverb of degree word	0.5	Mild, slight, light, relatively
	0.8	A little, a bit, some, somewhat
	1.2	More, so, more and more, comparatively
	1.25	Very, particularly, extraordinary, so much
	1.5	Excessively, too, much, overly
	2	Through and through, fully, most, completely

**Table 4:** Radiation radius of medical facilities.

Level of Medical Facilities	Radius of Radiation
Tertiary hospital	1000 m
Secondary hospital	800 m
Primary hospital	500 m
Community clinic	300 m
Drugstore	300 m
Fangcang shelter hospital	1500 m
COVID-19 designated hospital	1500 m

**Table 5:** Level classification criteria for the coordinated development of sentiment value to medical facility coverage ratio.

Coordinated Development Degree	Level	Scoring Standard
V1	High imbalance	0–0.100
V2	Serious imbalance	0.101–0.200
V3	Moderate imbalance	0.201–0.300
V4	Slight imbalance	0.301–0.400
V5	Approaching imbalance	0.401–0.500
V6	Bare coordination	0.501–0.600
V7	Primary coordination	0.601–0.700
V8	Moderate coordination	0.701–0.800
V9	Favourable coordination	0.801–0.900
V10	High coordination	0.901–1.000

**Table 6:** Presence of medical facilities in the three phases.

Stage	Medical Facilities
Stage A	Tertiary hospital, secondary hospital, primary hospital, community clinic, drugstore
Stage B	COVID-19-designated hospital, Fangcang shelter hospital, tertiary hospital, secondary hospital, primary hospital, community clinic, drugstore
Stage C	COVID-19-designated hospital, tertiary hospital, secondary hospital, primary hospital, community clinic, drugstore

## Data Availability

Not applicable.
